# Effect of early tumor response on the health-related quality of life among patients on second-line chemotherapy for advanced gastric cancer in the ABSOLUTE trial

**DOI:** 10.1007/s10120-020-01131-y

**Published:** 2020-11-02

**Authors:** Kazumasa Fujitani, Kohei Shitara, Atsuo Takashima, Keisuke Koeda, Hiroki Hara, Norisuke Nakayama, Shuichi Hironaka, Kazuhiro Nishikawa, Yutaka Kimura, Kenji Amagai, Hisashi Hosaka, Yoshito Komatsu, Ken Shimada, Ryohei Kawabata, Hideki Ohdan, Yasuhiro Kodera, Masato Nakamura, Takako Eguchi Nakajima, Yoshinori Miyata, Toshikazu Moriwaki, Tetsuya Kusumoto, Kazuo Nishikawa, Kazuhiro Ogata, Masashi Shimura, Satoshi Morita, Wasaburo Koizumi

**Affiliations:** 1 Department of Surgery, Osaka General Medical Center, 3-1-56, Bandaihigashi, Sumiyoshi-ku, Osaka, 558-0056 Japan; 2 Department of Gastrointestinal Oncology, National Cancer Center Hospital East, Kashiwa, Japan; 3 Gastrointestinal Medical Oncology Division, National Cancer Center Hospital, Tokyo, Japan; 4 Department of Medical Safety Science, Iwate Medical University, Morioka, Japan; 5 Department of Gastroenterology, Saitama Cancer Center, Ina-machi, Japan; 6 Department of Gastroenterology, Kanagawa Cancer Center, Yokohama, Japan; 7 Clinical Trial Promotion Department, Chiba Cancer Center, Chiba, Japan; 8 Department of Surgery, National Hospital Organization Osaka National Hospital, Osaka, Japan; 9 Department of Surgery, Sakai City Medical Center, Sakai, Japan; 10 Department of Gastroenterology, Ibaraki Prefectural Central Hospital, Kasama, Japan; 11 Division of Gastroenterology, Gunma Prefectural Cancer Center, Ohta, Japan; 12 Division of Cancer Chemotherapy, Hokkaido University Hospital Cancer Center, Sapporo, Japan; 13 Division of Medical Oncology, Department of Internal Medicine, Showa University Northern Yokohama Hospital, Yokohama, Japan; 14 Department of Surgery, Osaka Rosai Hospital, Osaka, Japan; 15 Department of Gastroenterological and Transplant Surgery, Graduate School of Biomedical and Health Sciences, Hiroshima University, Hiroshima, Japan; 16 Department of Gastroenterological Surgery, Nagoya University Graduate School of Medicine, Nagoya, Japan; 17 Aizawa Comprehensive Cancer Center, Aizawa Hospital, Nagano, Japan; 18 Department of Clinical Oncology, St. Marianna University School of Medicine, Kawasaki, Japan; 19 Kyoto Innovation Center for Next Generation Clinical Trials and iPS Cell Therapy, Kyoto University Hospital, Kyoto, Japan; 20 Department of Medical Oncology, Saku Central Hospital Advanced Care Center, Saku, Japan; 21 Division of Gastroenterology, Faculty of Medicine, University of Tsukuba, Tsukuba, Japan; 22 Department of Gastroenterological Surgery and Clinical Research Institute Cancer Research Division, National Kyushu Medical Center, Fukuoka, Japan; 23 Department of Medical Oncology and Hematology, Faculty of Medicine, Oita University, Oita, Japan; 24 Medical Affairs Department, Taiho Pharmaceutical Co., Ltd, Tokyo, Japan; 25 Data Science Department, Taiho Pharmaceutical Co., Ltd, Tokyo, Japan; 26 Department of Biomedical Statistics and Bioinformatics, Kyoto University Graduate School of Medicine, Kyoto, Japan; 27 Department of Gastroenterology, Kitasato University School of Medicine, Sagamihara, Japan

**Keywords:** Early tumor response, Health-related quality of life, Second-line chemotherapy, Paclitaxel, Advanced gastric cancer

## Abstract

**Background:**

This study evaluated the association between early tumor response at 8 weeks, previously reported as a positive outcome prognosticator, and health-related quality of life (HRQOL) in advanced gastric cancer (AGC) patients enrolled in the ABSOLUTE trial.

**Methods:**

HRQOL was assessed using the EuroQol-5 Dimension (EQ-5D) utility index score in patients with complete response (CR) + partial response (PR) and progressive disease (PD) at 8 weeks, and time-to-deterioration (TtD) of the EQ-5D score, with the preset minimally important difference (MID) of 0.05, was compared between these populations. Among the enrolled patients, 143 and 160 patients were assessable in weekly solvent-based paclitaxel (Sb-PTX) arm and weekly nanoparticle albumin-bound paclitaxel (nab-PTX) arm, respectively.

**Results:**

Changes of the EQ-5D score from baseline to 8 weeks in the nab-PTX arm were 0.0009 and − 0.1229 in CR + PR and PD patients, respectively; the corresponding values for the Sb-PTX arm were − 0.0019 and − 0.1549. For both treatments, changes of the EQ-5D score from baseline at 8 weeks were significantly larger in patients with PD than in those with CR + PR. The median TtD was 3.9 and 2.2 months in patients with CR + PR and PD, respectively, for nab-PTX [hazard ratio (HR) = 0.595, 95% confidence interval (CI) 0.358–0.989]. For Sb-PTX, the corresponding values were 4.7 and 2.0 months (HR = 0.494, 95% CI 0.291–0.841).

**Conclusions:**

Early tumor shrinkage was associated with maintained HRQOL in AGC patients on the second-line chemotherapy with taxanes.

## Introduction

Over 1,000,000 new cases of gastric cancer were reported worldwide in 2018. Globally, gastric cancer is the fifth most common malignancy and the third leading cause of cancer-related deaths [1]. In Japan, Gastric cancer is the second leading cause of death from cancer and the third most frequent cancer [2].

The prognosis of patients with advanced gastric cancer (AGC) is dismal. The standard treatment for AGC is chemotherapy, and fluoropyrimidine plus platinum remains the standard first-line chemotherapy [3–5]. Furthermore, until recently, second-line chemotherapy for patients with refractory disease or those who did not tolerate first-line chemotherapy was solvent-based (Sb) paclitaxel (PTX), docetaxel, and irinotecan [6–9]. However, Sb-PTX can cause hypersensitivity and anaphylactic reactions in some patients, which are likely related to polyethoxylated castor oil that is present in Sb-PTX [10, 11]. The 130 nm nanoparticle albumin-bound (nab) PTX formulation (Celgene, Summit, NJ, USA) has been developed to improve this treatment’s efficacy and minimize the risk of associated hypersensitivity without the use of premedication.

The ABSOLUTE trial has compared the efficacy and safety of nab-PTX administered every 1 or 3 weeks with weekly Sb-PTX in patients with AGC refractory to fluoropyrimidine-containing chemotherapy. The findings have confirmed non-inferiority of weekly nab-PTX vs. weekly Sb-PTX, measured with overall survival (OS), in contrast to Sb-PTX vs. nab-PTX every 3 weeks, which did not demonstrate similar performance. Moreover, patients’ health-related quality of life (HRQOL) was assessed in the ABSOLUTE trial with the EuroQol-5 Dimension questionnaire (EQ-5D). While patients treated with weekly nab-PTX and weekly Sb-PTX reported similar mean EQ-5D utility index scores over time, patients treated with tri-weekly nab-PTX reported lower scores than did patients in other treatment arms [12].

Preventing treatment-related complications and adverse events is an important consideration in clinical practice, alongside maintaining satisfactory HRQOL. Overall, physicians tend to believe that treatment efficacy and ability to maintain or improve HRQOL are correlated. In clinical practice, tumor-related symptoms in patients with tumor shrinkage generally improve. Therefore, improvement or maintenance of HRQOL based on treatment outcomes should be evaluated in cancer patients. Although HRQOL in various cancers has previously been studied in clinical trials, the association between HRQOL and disease progression has only been reported in breast, colorectal, and renal cancers [13]. Tumor shrinkage to chemotherapy in colorectal, lung, and renal cancer has been associated with the maintenance of satisfactory HRQOL and improvement of symptom extent and severity [14–17]. However, there have been few reports on these aspects in patients with AGC. Recently, clinically meaningful improvements of symptoms scores in patients with tumor shrinkage have been reported based on HRQOL assessments for AGC [18, 19]. However, there have been no studies for AGC that have examined the association between early tumor response and deterioration of comprehensive HRQOL. Thus, the aim of this study was to examine the association between early tumor response and HRQOL in post-hoc analysis of the ABSOLUTE trial data.

## Methods

### Study design and patients

The ABSOLUTE trial (Number JapicCTI-132059) was a randomized, open-label, non-inferiority, phase-3 trial conducted at 72 institutions in Japan. Patients were enrolled from March 2013 to May 2015. Patients with gastric cancer refractory to a first-line chemotherapy containing fluoropyrimidine were randomly allocated in 1:1:1 ratio to nab-PTX every 3 weeks at a dose of 260 mg/m^2^; nab-PTX at a dose of 100 mg/m^2^ delivered on days 1, 8, and 15 every 4 weeks; or Sb-PTX at a dose of 80 mg/m^2^ delivered on days 1, 8, and 15 every 4 weeks. Study treatment was continued until disease progression, unacceptable toxicity, or the emergence of other reasons for treatment discontinuation. The primary endpoint was overall survival (OS), estimated from the date of trial entry to the date of death from any cause or censored on the day of the last follow-up appointment. The secondary endpoints were progression-free survival (PFS), time to treatment failure, overall response rate (ORR), disease control rate, duration of response, dose intensity, safety, and quality of life (QOL). The institutional review board of each participating institution approved this trial, which was conducted according to the International Conference on Harmonization and Good Clinical Practice.

The results of the pre-planned analyses involved in this trial have been published previously [12]. Overall, 741 patients were randomly assigned to receive tri-weekly nab-PTX (*n* = 247), weekly nab-PTX (*n* = 246), or weekly Sb-PTX (*n* = 248). Of the 741 patients enrolled in this study, 469 patients (150 patients in the tri-weekly nab-PTX, 150 patients in the weekly nab-PTX, and 169 patients in the weekly Sb-PTX arm) had measurable lesions by computed tomography scanning or magnetic resonance imaging and data on a baseline assessment of the EQ-5D score.

In the present study, we focused on patients who had measurable lesions treated with weekly nab-PTX and Sb-PTX, and whose EQ-5D scores were available at baseline and at 8 weeks to assess the association between early tumor response and HRQOL. Patients treated with tri-weekly nab-PTX were excluded, as this treatment did not show non-inferiority to weekly Sb-PTX. Furthermore, these patients demonstrated a higher incidence of adverse events with the lowest mean EQ-5D utility index scores among the treatment arms. As a consequence, tri-weekly nab-PTX is not used for AGC in clinical practice.

### EuroQol-5D questionnaire

HRQOL was assessed using the validated Japanese version of the EQ-5D 3L, an international standardized questionnaire. The EQ-5D 3L questionnaire was collected at baseline and every 8 weeks during the first 24 weeks of the trial, and every 24 weeks thereafter. HRQOL assessments at 8 and 16 weeks were performed within ± 14 days and ± 28 days thereafter; a separate assessment was conducted immediately upon treatment discontinuation. The EQ-5D 3L comprises the following five items: ‘‘mobility,’’ ‘‘self-care,’’ ‘‘usual activities,’’ ‘‘pain/discomfort,’’ and ‘‘anxiety/depression,’’ which were assessed at three levels of description. The scores for each dimension were combined to obtain the overall EQ-5D health profile for each patient, consisting of a five-digit code. By applying weights derived from the general population, the health profiles were converted to the EQ-5D utility index score with a predetermined algorithm, where a score of 1 corresponded to full health, whereas a score of 0 represented poor health, considered equivalent to death [20]. Collected EQ-5D scores were converted to EQ-5D utility index score using the Japanese scoring algorithm [21]. Thus, higher EQ-5D utility index scores indicated better HRQOL. The mean EQ-5D utility index scores at baseline and at 8 weeks and changes of the mean EQ-5D utility index score from baseline to 8 weeks were examined.

### Grouping of patients by tumor response

Tumor response was assessed as complete response (CR), partial response (PR), stable disease (SD), or progressive disease (PD) according to the Response Evaluation Criteria in Solid Tumors guidelines, version 1.1. It was measured at 8 weeks by each investigator. We categorized eligible patients into four groups based on the treatment type and tumor response at 8 weeks: (1) patients enrolled in the weekly nab-PTX arm with CR or PR (CR + PR), (2) patients enrolled in the weekly nab-PTX arm with PD, (3) patients enrolled in the weekly Sb-PTX arm with CR or PR (CR + PR), and (4) patients enrolled in the weekly Sb-PTX arm with PD. Comparisons of changes of the mean EQ-5D utility index score were performed between patients whose overall response at 8 weeks was CR + PR and PD.

### Outcomes

In this study, the assessment for HRQOL comprised the comparison of mean EQ-5D utility index score between patients with CR + PR and PD at 8 weeks for each treatment arms and the estimation of time-to-deterioration (TtD) analyzed by minimally important difference (MID). MID refers to the smallest change in patient-reported outcomes that patients perceive as important, either beneficial or detrimental, indicating the extent of a clinically meaningful change of QOL between assessment points, which might lead the patient or clinician to consider a change in disease management [22]. MID was defined as the change in the EQ-5D utility index value of 0.05 from baseline to each assessment time point, based on previous reports [23]. The deterioration of HRQOL was defined as the decline of EQ-5D utility index score over MID. Only the first deterioration was counted as an event. Mortality was also counted as the event since the EQ-5D utility index score for death was specified to be 0. The remaining patients whose decline of EQ-5D utility index score was within MID were censored at the time of the final assessment. The effect of tumor response at 8 weeks on TtD was estimated in this study.

### Statistical analysis

This study was an exploratory analysis that was not pre-specified in the trial protocol. We performed all the analyses based on the data from patients who had measurable lesions and had the EQ-5D score assessed at baseline and at least once during the treatment. Confidence intervals (CIs) of between-group differences in the change of mean EQ-5D utility index score were calculated using the Greenwood’s formula. The Kaplan–Meier method was used to estimate the curves of TtD of patients with MID as events. The hazard ratios (HRs) and corresponding CIs for TtD were estimated using the Cox proportional hazards model. Given that these analyses were not pre-specified in the trial protocol, *p *values were not reported to prevent misinterpretations regarding efficacy; to evaluate comparisons, CIs were used instead of *p *values. All analyses were performed using SAS version 9.4.

## Result

### Patients’ characteristics

Of the 150 patients in the weekly nab-PTX arm, 143 patients had their EQ-5D score assessed at least once during treatment. In the weekly Sb-PTX arm, 160 out of 169 patients had their EQ-5D assessed at least once during treatment. Patients’ baseline demographic and clinical characteristics were similar between the treatment arms (Table 1). However, the duration of previous chemotherapy varied between the treatment arms.

**Table 1:** Baseline demographic and clinical characteristics of patients in the weekly nab-PTX arm and Sb-PTX arm

	nab-PTX (*N*=143)	Sb-PTX (*N*=160)
Age (years)		
Median, Range (Min, Max)	67.0 (29.0, 85.0)	66.0 (26.0, 88.0)
Sex, *n* (%)		
Male/Female	111 (77.6)/32 (22.4)	123 (76.9)/37 (23.1)
ECOG performance status, *n* (%)		
0/1/2	105 (73.4)/37 (25.9)/1 (0.7)	115 (71.9)/43 (26.9)/2 (1.3)
Histological type, *n* (%)		
Diffuse/Intestinal/Unknown	66 (46.2)/77 (53.8)/0 (0.0)	70 (43.8)/89 (55.6)/1 (0.6)
Previous gastrectomy, *n* (%)		
No/Yes	67 (46.9)/76 (53.1)	68 (42.5)/92 (57.5)
Number of organs with metastases, *n* (%)		
<2/≥2	66 (46.2)/77 (53.8)	62 (38.8)/98 (61.3)
Peritoneal metastasis (at randomization), *n* (%)		
No/Yes	88 (61.5)/55 (38.5)	96 (60.0)/64 (40.0)
Previous chemotherapy regimens, *n* (%)		
Fluoropyrimidine monotherapy	54 (37.8)	61 (38.1)
Doublet chemotherapy	77 (53.8)	87 (54.4)
Triplet chemotherapy	12 (8.4)	12 (7.5)
Duration of previous chemotherapy, *n* (%)		
<6 months/≥6 months	73 (51.0)/70 (49.0)	64 (40.0)/96 (60.0)
Type of treatment failure with previous chemotherapy, *n* (%)		
Adjuvant chemotherapy/first-line chemotherapy	39 (27.3)/104 (72.7)	46 (28.8)/114 (71.3)

*Nab-PTX* nanoparticle-bound paclitaxel, *Sb-PTX* solvent-based paclitaxel, *ECOG* Eastern Cooperative Oncology Group

### EQ-5D completion rate and mean utility index score at assessment times

**Fig. 1:**
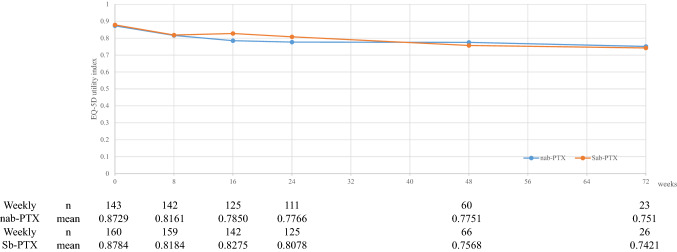
Mean EQ-5D utility index score at the assessment time points. *nab-PTX* nanoparticle-bound paclitaxel, *Sb-PTX* solvent-based paclitaxel

The EQ-5D completion rates are presented in Table 2. The EQ-5D completion rates were high in each treatment arm, and the EQ-5D completion rates at 8 and 16 weeks were 99.3 and 91.2%, respectively, in the weekly nab-PTX arm; they were 99.4 and 95.9%, respectively, in the weekly Sb-PTX arm. Although the EQ-5D completion rate decreased over time within each treatment arm, a high completion rate (> 90%) was maintained throughout 16 weeks in each treatment arm. The completion rates significantly decreased at 24 weeks and beyond in each treatment arm. The reasons for non-completion were treatment discontinuation due to disease progression, AEs, and missing EQ-5D scores. Between-assessment changes of EQ-5D utility index scores were similar in both treatment arms. (Fig. 1).

**Table 2:** EQ-5D completion rate among patients treated with weekly nab-PTX or Sb-PTX

Visit	Weekly nab-PTX	Weekly Sb-PTX
Baseline		
Expected to complete, *n*	143	160
Completed, *n*	143	160
Completion rate, %	100.0	100.0
Week 8		
Expected to complete, *n*	143	160
Completed, *n*	142	159
Completion rate, %	99.3	99.4
Week 16		
Expected to complete, *n*	137	148
Completed, *n*	125	142
Completion rate, %	91.2	95.9
Week 24		
Expected to complete, *n*	130	142
Completed, *n*	111	125
Completion rate, %	85.4	88.0
Week 48		
Expected to complete, *n*	79	91
Completed, *n*	60	66
Completion rate, %	75.9	72.5
Week 72		
Expected to complete, *n*	35	45
Completed, *n*	23	26
Completion rate, %	65.7	57.8
Week 96		
Expected to complete, *n*	12	11
Completed, *n*	10	8
Completion rate, %	83.3	72.7

Completion rate was defined as the proportion of patients who completed the EQ-5D score among those who were expected to complete it at each assessment, excluding those missing by death.

*nab-PTX* nanoparticle-bound paclitaxel, *Sb-PTX* solvent-based paclitaxel

### Assessment of tumor response and mean EQ-5D utility index score at 8 weeks

Of the 143 patients in the weekly nab-PTX arm and 160 patients in the weekly Sb-PTX arm, the HRQOL for one patient in each treatment arm was not evaluated because of missing EQ-5D score at 8 weeks. Among 142 patients in the weekly nab-PTX arm, 41 (28.9%), 68 (47.9%), and 33 (23.2%) patients had CR + PR, SD, and PD at 8 weeks, respectively. Likewise, of 159 patients in the weekly Sb-PTX arm, 29 (18.2%), 86 (54.1%), and 44 (27.7%) patients had CR + PR, SD, and PD at 8 weeks, respectively.

The mean EQ-5D utility index scores at baseline and at 8 weeks, and the changes of the mean EQ-5D utility index scores from baseline to 8 weeks for patients with CR + PR and PD in each treatment arm are shown in Table 3. The mean baseline EQ-5D utility index score for the weekly nab-PTX arm was 0.8516 and 0.8569 for patients with CR + PR and PD, respectively. The 95% CI of the difference in mean EQ-5D utility index scores at baseline between these populations was − 0.0758 to 0.0651, suggesting a small difference between CR + PR and PD patients. The mean baseline EQ-5D utility index score for the weekly Sb-PTX arm was 0.8593 and 0.8463 for patients with CR + PR and PD, respectively (95% CI in the difference between these populations, − 0.0559 to 0.0821). This result was similar to that of the weekly nab-PTX arm. There were no differences in the mean baseline EQ-5D utility index score between patients with CR + PR and PD, respectively, in each treatment arm, as the lower limit of the 95% CI for the difference was below 0. Moreover, the changes of the mean EQ-5D utility index score from baseline to 8 weeks in the weekly nab-PTX arm were 0.0009 and − 0.1229 in patients with CR + PR and PD, respectively (95% CI in the change difference between these populations, 0.0294–0.2182). The corresponding changes in the weekly Sb-PTX arm were − 0.0019 and − 0.1549 (95% CI in the change difference between these populations, 0.0507–0.2553). Overall, in each treatment arm, patients with CR + PR achieved a better score than did patients with PD. Differences in the changes of mean EQ-5D utility index scores from baseline to 8 weeks between patients with CR + PR and PD within each treatment arm were significant, as the corresponding 95% CI did not include 0. HRQOL assessment showed less deterioration in patients with CR + PR than in patients with PD at 8 weeks.

**Table 3:** Summary of the mean EQ-5D utility index score and the change from baseline at 8 weeks in the weekly nab-PTX arm and Sb-PTX arm

	Weekly nab-PTX (n=142)			Weekly Sb-PTX (n=159)		
	CR/PR	PD	CR/PR vs. PD 95% CI (min, max)^*^	CR/PR	PD	CR/PR vs. PD 95% CI (min, max)^*^
*n* (%) at 8 weeks	41 (28.9)	33 (23.2)		29 (18.2)	44 (27.7)	
Baseline, EQ-5D utility index score Mean (SD)	0.8516 (0.1417)	0.8569 (0.1620)	− 0.0758, 0.0651	0.8593 (0.1444)	0.8463 (0.1448)	− 0.0559, 0.0821
At 8 weeks, EQ-5D utility index score Mean (SD)	0.8525 (0.1915)	0.7340 (0.2247)	0.0220, 0.2150	0.8574 (0.1689)	0.6914 (0.2870)	0.0482, 0.2840
Change from baseline at 8 weeks in EQ-5D utility index score Mean (SD)	0.0009 (0.1639)	− 0.1229 (0.2422)	0.0294, 0.2182	− 0.0019 (0.1510)	− 0.1549 (0.2473)	0.0507, 0.2553

*nab-PTX* nanoparticle-bound paclitaxel, *Sb-PTX* solvent-based paclitaxel, *ORR* overall response rate, *SD* standard deviation, *CI* confidence interval

^*^Difference in the mean EQ-5D utility index score between baseline and each assessment time.

The effect of tumor response at 8 weeks on HRQOL deterioration with MID of 0.05 is shown in Table 4. In 11 of 41 patients with CR + PR (26.8%) in the weekly nab-PTX arm and in 7 of 29 patients with CR + PR (24.1%) in the weekly Sb-PTX arm, the changes of the mean EQ-5D utility index scores from baseline to 8 weeks exceeded the MID. Consequently, three-quarters of patients with CR + PR in both PTX arms demonstrated improvement or no change of HRQOL. In contrast, for 16 of 33 patients with PD (48.5%) in the weekly nab-PTX arm and for 24 of 44 patients with PD (54.5%) in the weekly Sb-PTX arm, the score change exceeded the MID. Approximately, half of the patients with PD in both PTX arms showed HRQOL deterioration.

**Table 4:** Association of tumor response at 8 weeks with the change of mean EQ-5D utility index score with MID of 0.05 in the weekly nab-PTX arm and Sb-PTX arm

	Weekly nab-PTX (*n* = 142)		Weekly Sb-PTX (*n* = 159)	
	CR/PR (*n* = 41)	PD (*n* = 33)	CR/PR (*n* = 29)	PD (*n* = 44)
Deterioration (over 0.05), *n* (%)	11(26.8)	16 (48.5)	7 (24.1)	24 (54.5)
No change or improvement (within 0.05), *n* (%)	30 (73.2)	17 (51.5)	22 (75.9)	20 (45.5)

Deterioration, patients whose the change of mean EQ-5D utility index score from baseline to 8 weeks exceeded MID; no change or improvement, patients whose the change of mean EQ-5D utility index from baseline to 8 weeks was within MID

*nab-PTX* nanoparticle-bound paclitaxel, *Sb-PTX* solvent-based paclitaxel, *MID* minimally important difference, *CR* complete response, *PR* partial response, *PD* progressive disease

### Time to deterioration of the EQ-5D utility index score analysis

**Fig. 2:**
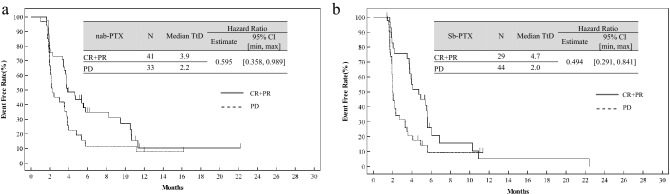
Kaplan–Meier curves for time-to-deterioration of the mean EQ-5D utility index score with MID of 0.05 as an event. **a** CR + PR vs. PD in weekly nab-PTX arm, **b** CR + PR vs. PD in weekly Sb-PTX arm. *TtD* time to deterioration, *nab-PTX* nanoparticle-bound paclitaxel, *Sb-PTX* solvent-based paclitaxel, *CR* complete response, *PR* partial response, *PD* progressive disease, *MID* minimally important difference

The median TtD of the mean EQ-5D utility index score in patients with CR + PR and PD was 3.9 and 2.2 months in the weekly nab-PTX arm and 4.7 and 2.0 months in the weekly Sb-PTX arm, respectively (Fig. 2a, b). The HRs for CR + PR vs. PD were 0.595 (95% CI 0.358–0.989) in the nab-PTX arm, and 0.494 (95% CI 0.291–0.841) in the Sb-PTX arm, respectively. The median TtD in patients with CR + PR was significantly longer than in patients with PD in both PTX arms.

## Discussion

The benefits of a new treatment or regimen can be evaluated based on improved OS and/or satisfactory HRQOL, which are metrics often used in clinical studies [24]. To the best of our knowledge, this is the first study to analyze the impact of early tumor response on the deterioration of comprehensive HRQOL among patients with AGC receiving the second-line chemotherapy. This exploratory analysis using the EQ-5D utility index score showed that tumor shrinkage to PTX treatment at 8 weeks was associated with lesser deterioration to the EQ-5D utility index score, suggesting that HRQOL was maintained in patients with tumor shrinkage. In addition, patients with CR + PR at 8 weeks were more likely to show longer TtD of their HRQOL than did patients with PD. These findings indicate that early tumor response at 8 weeks might be a reliable indicator of sustained maintenance of HRQOL in patients with AGC on second-line chemotherapy.

Previous studies rarely evaluated HRQOL in patients with AGC, as improved survival was prioritized as an outcome of interest. However, following improved survival due to the therapeutic effect of novel chemotherapy, HRQOL has recently become of interest in clinical trials on AGC. Associations between improvements in global HRQOL and treatments that confer clinical benefits (ORR, PFS and OS) in patients with AGC have been reported previously in several trials [8, 24–27]. HRQOL specific to cancer-related symptoms such as pain and appetite loss has also improved in AGC patients with tumor shrinkage due to chemotherapy [18, 19]. In the present study, we noted an association between tumor response at 8 weeks and patient-reported HRQOL. Compared to patients with PD in both PTX arms, patients with CR + PR, tumor shrinkage, at 8 weeks had a smaller change in their mean EQ-5D utility index score relative to baseline. Moreover, in both PTX arms, three-quarters of patients with CR + PR at 8 weeks did not demonstrate the decline of EQ-5D utility index score exceeding the MID, a clinically meaningful indicator of HRQOL deterioration. These findings coincided with the literature.

When analyzing the EQ-5D score in the SD + PD group, changes in the EQ-5D utility index score from baseline to 8 weeks in the weekly nab-PTX arm were 0.0009 and − 0.0790 in CR + PR and SD + PD patients, respectively, with 95% CI of the change difference between these populations being 0.0106–0.1493 (data not shown); the corresponding values for the weekly Sb-PTX arm were − 0.0019 and − 0.0736, respectively, with 95% CI of the change difference between these populations being − 0.0040 to 0.1475 (data not shown). These findings showed that HRQOL of patients with SD + PD at 8 weeks, compared to CR + PR, significantly deteriorated in the nab-PTX arm, but not in the Sb-PTX arm. Since the SD group comprises patients with tumor shrinkage and tumor growth, the impact of tumor shrinkage on HRQOL cannot be evaluated clearly in the SD group. Therefore, the SD group was not compared in this study.

We found that patients with tumor shrinkage at 8 weeks significantly prolonged median TtD which presented HRQOL-adjusted survival, compared to PD patients. Recently, early tumor shrinkage (ETS), defined as the percentage decrease in the sum of the target lesions’ longest diameters at 6–8 weeks, has been reported as a good predictor of OS, following the first-line chemotherapy for colorectal and gastric cancer [28–34]. Although the association between ETS and OS has not been previously reported for the second-line chemotherapy for AGC, our results suggested that ETS to the second-line chemotherapy in AGC patients might prolong OS. The improvement and maintenance of satisfactory HRQOL on ETS may be linked to better survival in AGC patients. The impact of HRQOL on OS is of interest because it may facilitate decision-making by physicians and patients regarding further treatment. In addition, the present study focused on early tumor response but not overall tumor response. If the patients and care-givers could anticipate the HRQOL as well as OS at an early stage of the second-line chemotherapy, it would give them a wide range of options for the remaining limited life.

This study has some limitations. First, this was an open-label study, which might have biased HRQOL assessment of patients during treatment. Second, HRQOL was assessed using the EQ-5D utility index score, in which each dimension was unable to be evaluated individually. The following items of HRQOL, such as nausea, vomiting, pain, and/or appetite loss, worsened with disease progression in the previous report estimated by QLQ-C30 [19]. Evaluation of HRQOL with cancer-specific tools like QLQ-C30 and QLQ-STO22 could have improved the validity of the present study. Third, some of the HRQOL data might have been obtained from patients treated with post-second-line chemotherapy agents, which could confound the results.

In conclusion, this is the first study to analyze the impact of early tumor response at 8 weeks on the deterioration of comprehensive HRQOL among patients with AGC receiving the second-line chemotherapy. Early tumor shrinkage was associated with maintained HRQOL in AGC patients on the second-line chemotherapy with taxanes.
