# Correction to: Effect of early tumor response on the health-related quality of life among patients on second-line chemotherapy for advanced gastric cancer in the ABSOLUTE trial

**DOI:** 10.1007/s10120-020-01144-7

**Published:** 2020-12-09

**Authors:** Kazumasa Fujitani, Kohei Shitara, Atsuo Takashima, Keisuke Koeda, Hiroki Hara, Norisuke Nakayama, Shuichi Hironaka, Kazuhiro Nishikawa, Yutaka Kimura, Kenji Amagai, Hisashi Hosaka, Yoshito Komatsu, Ken Shimada, Ryohei Kawabata, Hideki Ohdan, Yasuhiro Kodera, Masato Nakamura, Takako Eguchi Nakajima, Yoshinori Miyata, Toshikazu Moriwaki, Tetsuya Kusumoto, Kazuo Nishikawa, Kazuhiro Ogata, Masashi Shimura, Satoshi Morita, Wasaburo Koizumi

**Affiliations:** 1 Department of Surgery, Osaka General Medical Center, 3-1-56, Bandaihigashi, Sumiyoshi-ku, Osaka, 558-0056 Japan; 2 Department of Gastrointestinal Oncology, National Cancer Center Hospital East, Kashiwa, Japan; 3 Gastrointestinal Medical Oncology Division, National Cancer Center Hospital, Tokyo, Japan; 4 Department of Medical Safety Science, Iwate Medical University, Morioka, Japan; 5 Department of Gastroenterology, Saitama Cancer Center, Ina-machi, Japan; 6 Department of Gastroenterology, Kanagawa Cancer Center, Yokohama, Japan; 7 Clinical Trial Promotion Department, Chiba Cancer Center, Chiba, Japan; 8 Department of Surgery, National Hospital Organization Osaka National Hospital, Osaka, Japan; 9 Department of Surgery, Sakai City Medical Center, Sakai, Japan; 10 Department of Gastroenterology, Ibaraki Prefectural Central Hospital, Kasama, Japan; 11 Division of Gastroenterology, Gunma Prefectural Cancer Center, Ohta, Japan; 12 Division of Cancer Chemotherapy, Hokkaido University Hospital Cancer Center, Sapporo, Japan; 13 Division of Medical Oncology, Department of Internal Medicine, Showa University Northern Yokohama Hospital, Yokohama, Japan; 14 Department of Surgery, Osaka Rosai Hospital, Osaka, Japan; 15 Department of Gastroenterological and Transplant Surgery, Graduate School of Biomedical and Health Sciences, Hiroshima University, Hiroshima, Japan; 16 Department of Gastroenterological Surgery, Nagoya University Graduate School of Medicine, Nagoya, Japan; 17 Aizawa Comprehensive Cancer Center, Aizawa Hospital, Nagano, Japan; 18 Department of Clinical Oncology, St. Marianna University School of Medicine, Kawasaki, Japan; 19 Kyoto Innovation Center for Next Generation Clinical Trials and iPS Cell Therapy, Kyoto University Hospital, Kyoto, Japan; 20 Department of Medical Oncology, Saku Central Hospital Advanced Care Center, Saku, Japan; 21 Division of Gastroenterology, Faculty of Medicine, University of Tsukuba, Tsukuba, Japan; 22 Department of Gastroenterological Surgery and Clinical Research Institute Cancer Research Division, National Kyushu Medical Center, Fukuoka, Japan; 23 Department of Medical Oncology and Hematology, Faculty of Medicine, Oita University, Oita, Japan; 24 Medical Affairs Department, Taiho Pharmaceutical Co., Ltd, Tokyo, Japan; 25 Data Science Department, Taiho Pharmaceutical Co., Ltd, Tokyo, Japan; 26 Department of Biomedical Statistics and Bioinformatics, Kyoto University Graduate School of Medicine, Kyoto, Japan; 27 Department of Gastroenterology, Kitasato University School of Medicine, Sagamihara, Japan

## Correction to: Gastric Cancer 10.1007/s10120-020-01131-y

The article “Effect of early tumor response on the health-related quality of life among patients on second-line chemotherapy for advanced gastric cancer in the ABSOLUTE trial”, written by Kazumasa Fujitani, Kohei Shitara, Atsuo Takashima, Keisuke Koeda, Hiroki Hara, Norisuke Nakayama, Shuichi Hironaka, Kazuhiro Nishikawa, Yutaka Kimura, Kenji Amagai, Hisashi Hosaka, Yoshito Komatsu, Ken Shimada, Ryohei Kawabata, Hideki Ohdan, Yasuhiro Kodera, Masato Nakamura, Takako Eguchi Nakajima, Yoshinori Miyata, Toshikazu Moriwaki, Tetsuya Kusumoto, Kazuo Nishikawa, Kazuhiro Ogata, Masashi Shimura, Satoshi Morita & Wasaburo Koizumi, was originally published electronically on the publisher’s internet portal on 02 November 2020 without open access. With the author(s)’ decision to opt for Open Choice the copyright of the article changed on 09 November 2020 to © The Authors 2020 and the article is forthwith distributed under a Creative Commons Attribution 4.0 International License, which permits use, sharing, adaptation, distribution and reproduction in any medium or format, as long as you give appropriate credit to the original author(s) and the source, provide a link to the Creative Commons licence, and indicate if changes were made.

The images or other third party material in this article are included in the article’s Creative Commons licence, unless indicated otherwise in a credit line to the material. If material is not included in the article’s Creative Commons licence and your intended use is not permitted by statutory regulation or exceeds the permitted use, you will need to obtain permission directly from the copyright holder.

To view a copy of this licence, visit http://creativecommons.org/licenses/by/4.0.

The original article has been updated.

